# Discriminating bovine mastitis pathogens by combining loop-mediated isothermal amplification and amplicon-binding split trehalase assay

**DOI:** 10.3389/fvets.2024.1389184

**Published:** 2024-06-03

**Authors:** Zhuohan Miao, Jeroen De Buck

**Affiliations:** Faculty of Veterinary Medicine, University of Calgary, Calgary, AB, Canada

**Keywords:** bovine mastitis, split trehalase, loop-mediated isothermal amplification, bacteria, Gram-type

## Abstract

Bovine mastitis is predominantly caused by intramammary infections with various Gram-positive and Gram-negative bacteria, requiring accurate pathogen identification for effective treatment and antimicrobial resistance prevention. Here, a novel diagnostic method was developed to detect mastitis pathogens in milk samples by combining loop-mediated isothermal amplification with a split enzyme biosensor whereby trehalase fragments were fused with a DNA-binding protein, SpoIIID. Three primer sets, LAMPstaph, LAMPstrep, and LAMPneg, harboring SpoIIID recognition sequences targeted *Staphylococcus, Streptococcus*, and Gram-negative pathogens, respectively. Limits of detection were determined for DNA extracted from bacterial culture and bacteria-spiked milk. The combined method detected as low as 2, 24, and 10 copies of genomic DNA of staphylococci, streptococci and *Escherichia coli* and 11 CFU/ml for milk spiked with *Escherichia coli*. Higher detection limits were observed for Gram-positive bacteria in spiked milk. When testing genomic DNA of 10 mastitis isolates at concentrations of 10^6^ and 10^3^ copies per reaction, no cross-reactivity was detected for LAMPstaph nor LAMPstrep, whereas the LAMPneg assay cross-reacted only with *Corynebacterium* sp. at the highest concentration. This combined method demonstrated the potential to distinguish mastitis pathogenic Gram types for a rapid decision of antimicrobial treatment without culturing.

## 1 Introduction

Bovine mastitis, indicating inflammation of the mammary gland tissue, poses a significant challenge to the global dairy industry (1). Characterized as either clinical or subclinical mastitis (2), this condition is predominantly caused by intramammary infections (IMI) by a range of bacterial species, including Gram-positive and Gram-negative varieties which can be further categorized into contagious [e.g., *Staphylococcus aureus* (*S. aureus*), *Streptococcus agalactiae* (*S. agalactiae*)] and environmental [e.g., *Escherichia coli* (*E. coli*), non-aureus staphylococci (NAS), *Streptococcus uberis* (*S. uberis*)] bacteria or both [e.g., *Streptococcus dysgalactiae* (*S. dysgalactiae*)] (3, 4). Key pathogens for clinical mastitis are *E. coli, S. aureus, S. agalactiae* and *S. dysgalactiae* (5–8). Subclinical mastitis is primarily caused by minor pathogens like NAS and *Corynebacterium bovis* (9, 10). *S. aureus*, as the most frequent contagious pathogens, chronically infects the mammary gland with the production of degradative enzymes and toxins that damage the udder tissue irreversibly. Acting as an opportunistic pathogen, *E. coli* induces lasting harm to mammary gland tissue through the release of endotoxin, such as lipopolysaccharide (LPS). Additionally, environmental etiological agents such as *S. uberis* and *Enterococcus* spp. have been reported to recurrently instigate mastitis by forming biofilms (11, 12).

To minimize economic losses associated with bovine mastitis, accurate identification of pathogen Gram types is of critical importance to link the cause with the appropriate antimicrobial treatment (13). Mastitis diagnosis involves assessing disease indicators and identifying the causative bacteria (14). Pathogen identification is still commonly done by culture-based methods, involving culturing milk samples on suitable media, counting colony forming units (CFUs), and assessing colony phenotypes, which hold the possibility to assess antibiotic sensitivity on the isolates and aid therapeutic intervention. However, challenges related to time-to-result and on-farm implementation restrict the widespread use of culture-based methods (15). Molecular amplification accelerates the precise identification of mastitis pathogens and is most commonly done by PCR. Compared with conventional culture methods, PCR analysis consistently demonstrated higher specificity and sensitivity to detect the major pathogens as well as the NAS and *Corynebacterium bovis* in a large number of clinic and sub-clinic samples (16, 17). However, utilizing loop-mediated isothermal amplification (LAMP) as a component of on-farm diagnostics could be more promising, given its ability to detect genetic targets in under 30 min and higher sensitivity than PCR with a more simplified sample preparation (18–20). Positive results can be observed by changes in fluorescence or turbidity, and lateral flow assay (21–23).

A novel strategy for the detection of mastitis causative pathogens in milk samples involves combining the split trehalase biosensing platform with DNA-binding protein specificity and LAMP amplicons containing protein-binding sites. The periplasmic trehalase (TreA) can be experimentally split into inactive complementary N and C domains and the trehalase activity can be conditionally restored upon incubation with specific substrates (24, 25). Glucose, the main product of this restoration, can be quantified using a colorimetric assay or a handheld glucometer (25). Various diagnostic assays utilize split TreA fusion proteins to detect biomarkers, including immunoglobulin G, blood calcium, and anti-bovine leukemia virus antibodies (26–28). By incorporating a specific protein-binding sequence into PCR primers, the Gram-types of mastitis pathogens were discriminated in previous research (29). This application incorporates the high-affinity binding of SpoIIID monomer, a sporulation transcriptional regulator from *Bacillus subtilis*, to a consensus sequence 5′-TAGGACAAGC-3′- (30).

This study aims to employ split TreA-SpoIIID fusion proteins to identify the Gram-type of IMI organism by recognizing SpoIIID-binding sites incorporated in Gram-specific LAMP products. For this purpose, three LAMP primer sets with incorporated SpoIIID recognition sequences were successfully designed, targeting *Staphylococcus, Streptococcus*, and Gram-negative pathogens.

## 2 Materials and methods

### 2.1 Bacterial strain

The bacterial strains used in this study are *Staphylococcus devriesei* 41816325, *Streptococcus uberis* 10501290, *Streptococcus dysgalactiae* 20304478, *Streptococcus uberis* 10107041, *Corynebacterium amycolatum* 40200255, *Escherichia coli* 10109298, *Klebsiella pneumoniae* 10116692 and *Enterobacter hormaechei* 11104490 isolates from Canadian Bovine Mastitis Research Network (Montreal, QC, Canada), *Staphylococcus aureus* USA300 (ATCC; Manassas, VA, USA) and *Escherichia coli* DH10β (New England Biolabs; Whitby, ON, Canada). Isolates were maintained as glycerol stocks and cultivated in Brain Heart Infusion (BHI) broth (BD; Mississauga, ON, Canada). Single colonies were picked from Tryptic Soy Agar (TSA) plates with 5% sheep blood (Hardy Diagnostics; Santa Maria, CA, USA) after the incubation at 37°C overnight.

### 2.2 Oligonucleotides

DG74 is a universal primer for the broad-range amplification of Gram-positive (Gram+) and Gram-negative (Gram-) bacteria (31) (Table 1). Two SpoIIID recognition sequence were added in tandem as an extension to 5′ end of DG74 to form FF_DG74, which was hybridized with its reverse complementary oligonucleotide through a thermal cycling program: heating at 95°C for 7 min, equilibrating at 56°C for 5 min, and cooling down to 25°C for 5 min to form double-stranded dsFF_DG74.

**Table 1:** List of oligonucleotides^*†^.

**Name**	**Sequence (5^′^-3^′^-)**
DG74^‡^	AGGAGGTGATCCAACCGCA
FF_DG74	ATTAGGACAAGCTTTTTAGGACAAGCAGGAGGTGATCCAACCGCA
**LAMP primers for** ***Staphylococcus*** **and** ***Streptococcus*** **(LAMPstaph and LAMPstrep)**	
F3	TGCCCCTTATGAYYTGG
B3	GAACGTATTCACCGYR
FIP_staph	TTTGCWTGACCTCGCGGCTACACACGTGCTACAATG
BIP_staph	TCCCATAAAGTTGTTCTCAGTTCGGCGATTACTAGCGATTCCAGCTTC
FIP_strep	TTAGCTTGCCGTCACCGCTACRCACGTGCTACAATG
BIP_strep	TCTCTTAAAGCCAATCTCAGTTCGGCGATTACTAGCGATTCCGACTTC
LF_Ext_staph	ATTAGGACAAGCTTTTTAGGACAAGCTTTMGCTGCCCTTTGTATTGT
LF_Ext_strep	ATTAGGACAAGCTTTTTAGGACAAGCGCTTGCGACTCGTTGTACCAA
LB_Ext	ATTAGGACAAGCTTTTTAGGACAAGCTAGKCTGCAACTCGMCTACA
**LAMP primers for Gram-negative Bacteria (LAMPneg)**	
F3_neg	TGGGATTAGCTWGTWGGTG
B3_neg	TTCAYACACGCGGCATG
FIP_neg	AGTGTGGCTGGTCATCCTCGGTAACGGCTCACCW
BIP_neg	GGAACTGAGACACGGTCCAGCTGCATCAGGCTTG
LF_Ext_neg	ATTAGGACAAGCTTTTTAGGACAAGCAGACCAGCTAGGGATCGTCG
LB_Ext_neg	ATTAGGACAAGCTTTTTAGGACAAGCTGGGGAATATTGCACAATGGGC

^*^FF, two linked SpoIIID recognition sequences; F3, forward outer primer; B3, backward outer primer; FIP, forward inner primer; BIP, backward inner primer; LF, forward loop primer; LB, backward loop primer; Ext., extension to loop primer; staph, staphylococci; strep, streptococci; neg., Gram-negative bacteria. ^†^Five degenerate bases used in thirteen positions (Y = C/T, R = A/G, W = A/T, M = A/C and K = G/T). ^‡^Primer as described by previous study (31).

The primer sets for LAMP assays were designed using the NEB LAMP primer design tool (https://lamp.neb.com/#!/) with 16S ribosomal RNA genes of *Staphylococcus* (*S. cohnii, S. aureus, S. arlettae*), *Streptococcus* (*S. uberis, S. dysgalactiae, S. agalactiae*), and Gram-negative (*Klebsiella, Enterobacter, E. coli*) strains from GenBank [Sequence ID: OP847727.1, CP127827.1, MN851074.1, MK330595.1, LC317295.1, HQ180246.1, JQ837267.1, AB609046.1, and AF403733.1] (Table 1). Multiple parameters were manually modified to preferred values following the basic primer design guidelines recommended by the Eiken genome site (https://loopamp.eiken.co.jp/en/lamp/0202.html), followed by a putative secondary structure check with IDT OligoAnalyzer (https://www.idtdna.com/calc/analyzer). All the gene alignment analyses were completed with Geneious (version 9.0.5). The specificity of primer to organism was checked with the nucleotide BLAST tool (https://blast.ncbi.nlm.nih.gov/Blast.cgi).

### 2.3 DNA extraction

The genomic DNA (gDNA) from bacterial cultures was extracted with the DNeasy Blood & Tissue Kit (QIAGEN; Toronto, ON, Canada) following the manufacturer's protocols. Briefly, the bacterial overnight culture was centrifuged at 17, 900 *g* for 5 min. The cell pellet was resuspended in 1 × PBS followed by the addition of 20 μl of proteinase K and 200 μl of Buffer AL and incubation at 56°C for 30 min. For DNA extraction from Gram-positive bacterial cultures, 0.5 g of 0.1- and 0.5-mm silica beads (Zymo Research; Irvine, CA, USA) was added to the lysate and submitted to the bead beater (BioSpec Products; Bartlesville, OK, USA) for two 5-min rounds, followed by centrifugation at 16, 000 *g* for 5 min to collect the supernatant before mixing with absolute ethanol. The thoroughly mixed solution was applied to the DNeasy spin column and experienced the washing steps with Buffer AW1 and AW2. 50 μl of DNase-free water was pipetted onto the DNeasy membrane to elute DNA by centrifugation at 6, 000 *g* for 1 min.

Pasteurized milk was purchased from a local retail outlet. Two ml of milk aliquot spiked with bacterial culture were centrifuged at 6, 700 *g* for 10 min. The pellet was resuspended in sterile 0.9% NaCl solution, centrifuged at 6, 700 *g* for 5 min, resuspended in TENS buffer [10 mM Tris-HCl (pH 8.0), 1 mM EDTA, 150 mM NaCl, 0.5% SDS], and incubated with proteinase K (1.5 mg/ml) at 56°C for 1 h on a heating block without shaking (32). Lysate was then bead-beaten by silica beads (0.5 g), and centrifuged at 12, 000 *g* for 10 min. The collected supernatant was mixed with an equal volume of phenol: chloroform: isoamyl alcohol (25:24:1) and centrifuged again. The upper aqueous phase was mixed with an equal volume of chloroform: isoamyl alcohol (24:1), and centrifuged again, where the harvested aqueous layer was added with 100% 2-propanol and centrifuged again. Pellet was resuspended in ice-cold 100% ethanol, stored at −80°C for 30 min and centrifuged at 20, 000 *g* for 20 min. The resulting DNA pellet was washed with room temperature (RT) 70% ethanol, centrifuged at 12, 000 *g* for 10 min, air-dried and eluted in DNase-free water (Thermo Fisher; Ottawa, ON, Canada). All the centrifugation was performed at RT.

### 2.4 Purification of recombinant proteins

Expression and purification of recombinant proteins N-SpoIIID and SpoIIID-C were performed following the Ni-NTA affinity chromatography method describing in our previous study (29). Briefly, induced cell lysates were sonicated in denaturing conditions and loaded onto the ÄKTA Go system (Cytiva; Marlborough, MA, USA). The eluted recombinant proteins were gradually refolded by dialysis against Snakeskin™ membrane (Thermo Fisher; Ottawa, ON, Canada) in sodium maleate buffer (50 mM, pH 6) for 24 h at 4°C.

### 2.5 Recombinant protein-DNA binding detection assay

The set-up of the recombinant protein-DNA binding detection assay was previously described (29). Recombinant proteins N-SpoIIID (20 kDa) and SpoIIID-C (65 kDa) were adjusted to a 1:1 molar ratio in a 150-μl reaction containing 1.5 μg N-SpoIIID, 5 μg of SpoIIID-C and 250 mM trehalose. Glucose oxidase, horseradish peroxidase and o-dianisidine were added at respective final concentrations of 0.1 mg/ml, 0.2 U/ml and 0.12 mg/ml to each colorimetric assay (three-enzyme assay or 3EA), which was incubated for 90 min at R/T and measured the OD_450_ value every minute to determine the glucose level. Positive control was conducted with dsFF_DG74 at the optimized 200 nM as DNA substrate which contains the tandem-linked SpoIIID recognition fragments. DNase-free water served as the non-template control. This enzyme colorimetric assay was also named as Amplicon Binding Split Trehalase assay (ABSTA).

### 2.6 Loop-mediated isothermal amplification

The LAMP assays in this study were performed with the WarmStart™ LAMP kit (New England Biolabs; Whitby, ON, Canada). Each LAMP primer set was prepared in the ratio specified by the manufacturer's protocols. A 25-μl reaction was subjected to the CFX96 Real-Time PCR detection system (Bio-Rad; Mississauga, ON, Canada) programmed to keep the temperature at 65°C for 30 min. The resulting LAMP product (5 μl) was submitted to 1% agarose gel electrophoresis. 5 μl of LAMP product was submitted to the ABSTA. Both LAMP assay and the ABSTA were performed in triplicate.

### 2.7 Limit of detection

For bacterial cultures, the extracted gDNA of *S. devriesei, E. coli* DH10β and *S. uberis* was measured by NanoPhotometer™ NP80 UV/Vis Spectrophotometer (Implen; Westlake Village, CA, USA) and adjusted to 50 ng/μl and then 10-fold serially diluted with DNase-free water to seven dilutions (10^−1^ to 10^−7^). Per LAMP reaction, 50 to 5 × 10^−6^ ng of gDNA of each bacteria was analyzed with the corresponding primer set. DNase-free water was used in the non-template control. The gDNA copy number for each reaction was calculated based on the genome length of *S. devriesei* [GenBank ID: GCA_900458355.1], *S. uberis* [GCF_900460135.1], and *E. coli* DH10b (33), respectively. The detection limit of ABSTA was defined as the gDNA copy number where the ABSTA endpoint signal level became significantly different from the LAMP non-template control.

For bacterial DNA in milk samples, bacterial cultures of *S. devriesei, E. coli* DH10β and *S. uberis* with OD_600_ of 1 were serially 10-fold diluted with 1 × PBS, where the bacteria were further diluted a hundred fold after transferring 18 μl of dilution to 1.8 ml of milk aliquot. Two additional milk aliquots spiked with 200 μl of undiluted cultures were set up for *S. devriesei* and *S. uberis*, respectively. In parallel, 100 μl of each bacterial culture dilution was plated to count the CFU. DNA extracted from 1 × PBS-spiked milk was used in LAMP negative control in addition to the LAMP non-template control with DNase-free water. The limit of detection was defined as the bacterial CFU per milliliter (CFU/ml) in milk samples when the ABSTA signal level became significantly different from the PBS-spiking control.

The above-mentioned DNA preparations from milk samples spiked with Gram-positive bacteria (*S. devriesei*) and Gram-negative bacteria (*E. coli*) were also subjected to PathoProof™ Mastitis Complete-16 Kits (Thermo Fisher; Vantaa, Uusimaa, Finland). DNA of sixteen bacteria can be targeted by four primer mixes, each of them detecting four different bacteria and one universal amplification standard. This multiplex real-time PCR was conducted in the CFX96 detection system following the thermal cycling program: 95°C for 10 min (segment 1, 1 cycle); 95°C for 5 sec, 60°C for 1 min, endpoint read (segment 2, 40 cycles). DNA extracted from PBS-spiked milk samples, the PathoProof™ Universal Amplification Standard, and DNase-free water were utilized as the extraction control, positive control, and negative control of this real-time PCR, respectively. *Ct* values below 37 were reported as positive results.

### 2.8 Specificity of detection by cross-reactivity test

The specificity was defined as the analytical specificity of LAMP determined using the gDNA of 10 isolates of *S. devriesei, S. aureus, S. uberis, S. dysgalactiae, Corynebacterium amycolatum, E. coli, Klebsiella pneumoniae*, and *Enterobacter hormaechei*. The gDNA of each strain was prepared to 10^6^ and 10^3^ genomic copies to be added to two individual LAMP reactions conducted with the same primer set, respectively. High specificity corresponds with the absence of false-positive amplification by each primer set at both copy numbers of the above-mentioned isolates.

### 2.9 Statistical analyses

One-way ANOVA and Tukey multiple comparisons were performed on three-enzyme assay results of groups corresponding to the LAMP products using the same primer set. All the analyses were performed using R v4.2.0 (RStudio; Boston, MA, USA) and were considered statistically significant when *p* < 0.05.

## 3 Results

### 3.1 Design of Gram type-specific LAMP reactions and amplicon binding split trehalase assay

**Figure 1:**
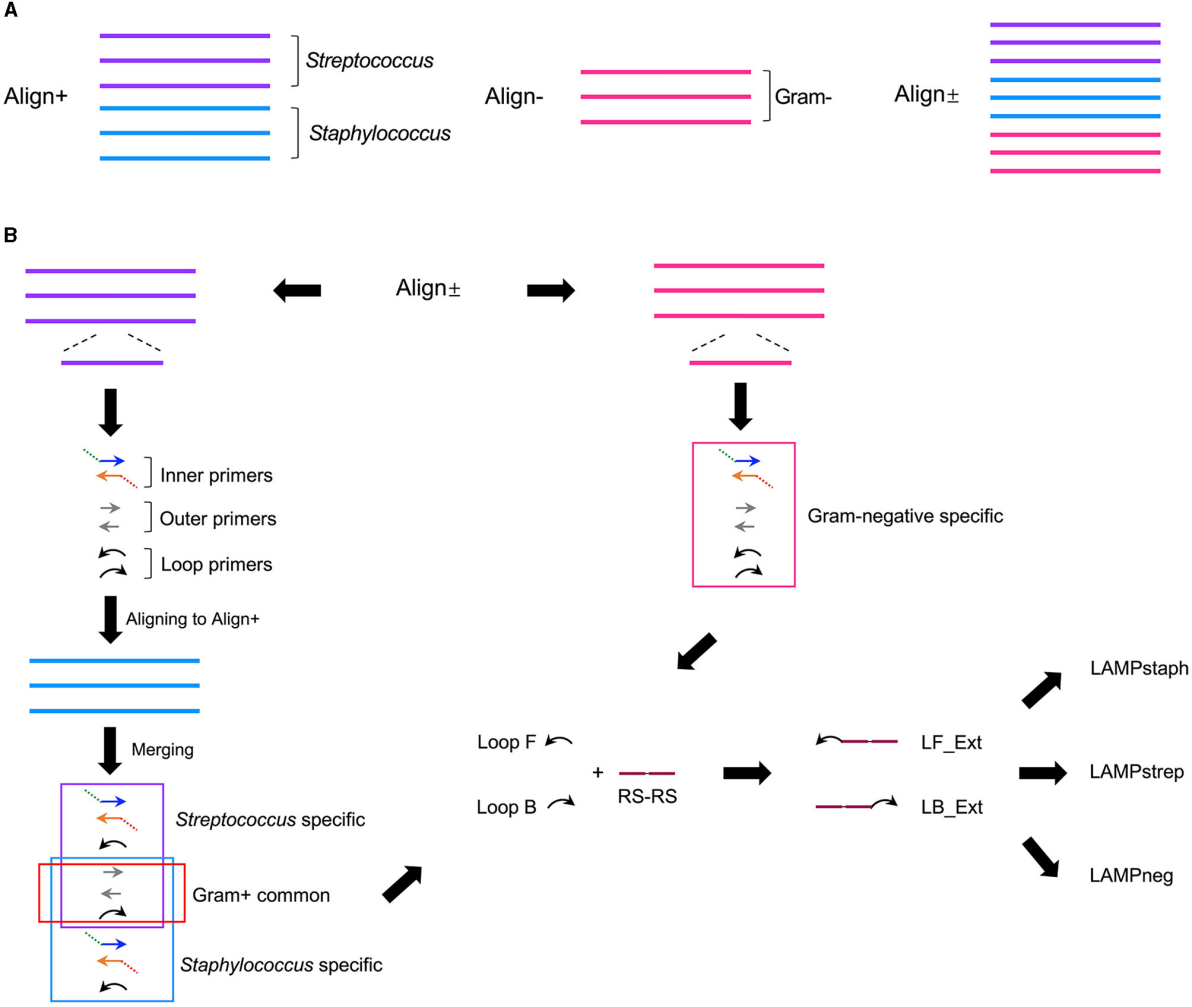
Schematic of design process of LAMP primer sets specific for *Staphylococcus, Streptococcus* and Gram-negative bacteria. **(A)** 16S rRNA genes from three staphylococci strains (blue line) and three streptococci strains (purple line) were aligned as Align+, and from three Gram-negative strains (pink line) were aligned as Align-. Align+ and Align- was combined as Align±. **(B)** A *Streptococcus* specific LAMP primer set (purple box) containing paired inner, outer, and loop primers, was generated by the homologous sequence in streptococci alignment in Align± and aligned to staphylococci alignment in Align+ to generate *Staphylococcus* specific primer set (blue box) with three Gram-positive primers (red box) shared by both sets. A Gram-negative specific primer set (pink box) was generated by the conserved sequence selected from Align- in the Align±. Tandem SpoIIID recognition sequences (RS-RS) (plum line) were fused to 5′ end of loop primer forward (Loop F) and backward (Loop B) from the above three primer sets to generate extended loop primer pairs (LF_Ext and LB_Ext), resulting in three modified LAMP primer sets, LAMPstaph, LAMPstrep and LAMPneg.

**Figure 2:**
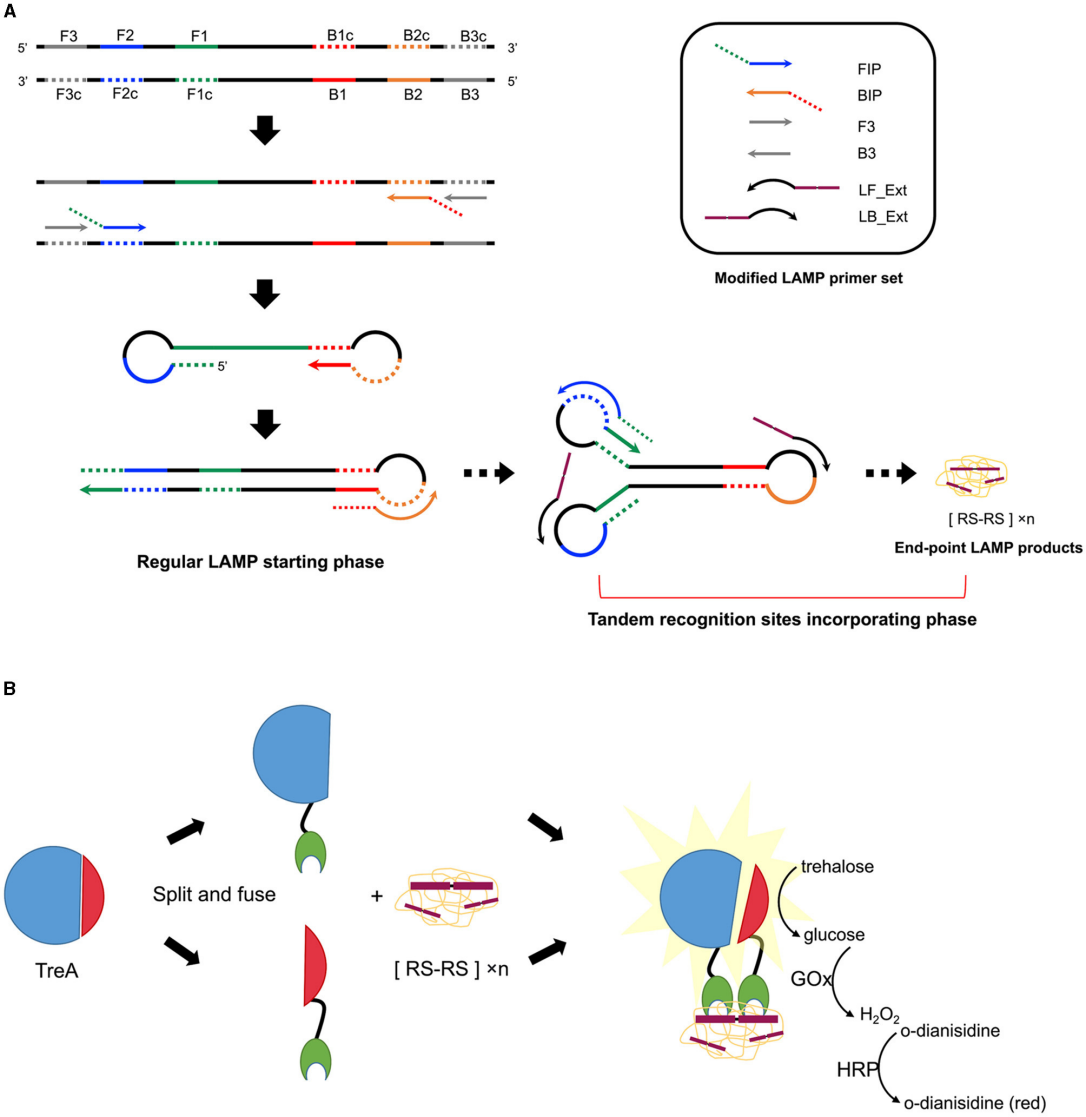
Schematic of the incorporation of tandem SpoIIID recognition sites into LAMP products followed by ABSTA. **(A)** Modified LAMP reaction. Colored regions F1 (green), F2 (blue), F3 (gray), B1 (red), B2 (orange), and B3 (gray) on the target DNA are shown in solid lines, with complementary regions indicated by dashed lines. Inner primers are fused either by sequences of F1c and F2 (FIP) or by B1c and B2 (BIP). Outer primers F3 and B3 hybridize F3c and B3c regions, respectively. Arrowheads on primers represent the 3′–end. Following the regular LAMP starting phase, extended loop primers (LF_Ext and LB_Ext) hybridize the region between F1 and F2, and B1 and B2 on intermediate concatemer amplicons, incorporating abundant tandem recognition sites ([RS-RS] × n) onto double-stranded loops in the end-point products mix. **(B)** ABSTA detection relies on the fusion of split trehalase (TreA) fragments (TreAC in blue and TreAN in red) with protein SpoIIID (green). Binding of SpoIIID to recognition sites incorporated in LAMP products brings TreAC and TreAN together, restoring trehalase activity. Glucose produced from trehalose is then measuring using a colorimetric enzyme reaction involving glucose oxidase (GOx), horse radish peroxidase (HRP), and o-dianisidine.

Six Gram-positive (*S. cohnii, S. aureus, S. arlettae, S. uberis, S. dysgalactiae, S. agalactiae*) and three Gram-negative bacteria (*Klebsiella pneumoniae, Enterobacter hormaechei, E. coli*) 16S rRNA genes were aligned separately or combined, resulting in three alignments named Align+, Align- and Align± (Figure 1A). A 280-bp length of homologous sequence was selected from the sub-alignment of *Streptococcus* 16S genes and generated the *Streptococcus*-specific primer set. The highly conservative forward external primer (F3), backward external primer (B3) and loop primer backward (LB) were reused in the *Staphylococcus* set which contained three more *Staphylococcus*-specific primers (Figure 1B). No consensus identity with Gram-negative genes was detected for the two Gram-positive primer sets. The Gram-negative-specific primer set was generated directly from the selected homologous region of Align- with no consensus to Align+. Two SpoIIID recognition sequences were incorporated in tandem at the 5′ ends of all the loop primers to obtain three Gram-type specific primer sets, namely LAMPstaph (F3, B3, FIP_staph, BIP_staph, LF_Ext_staph, LB_Ext), LAMPstrep (F3, B3, FIP_strep, BIP_strep, LF_Ext_strep, LB_Ext) and LAMPneg (F3_neg, B3_neg, FIP_neg, BIP_neg, LF_Ext_neg, LB_Ext_neg) (Table 1, Figure 2A). Each primer against 100 subject sequences of the corresponding organisms in NCBI nucleotide database showed matches with 100% identity over the entire sequence length, after considering the degenerate bases. In the proposed LAMP reaction, the increasing hybridization of loop primers during the strand displacement DNA synthesis phase was expected to produce an abundance of double-stranded SpoIIID recognition sites in the endpoint amplicons. When encountering these LAMP products, split trehalase fusion proteins SpoIIID-C and N-SpoIIID will be complemented due to the proximity achieved by binding to the recognition sites present in the amplicons, resulting in restored trehalase activity and quantifiable glucose production (Figure 2B).

### 3.2 Detection limit of ABSTA following LAMP with bacterial genomic DNA

**Figure 3:**
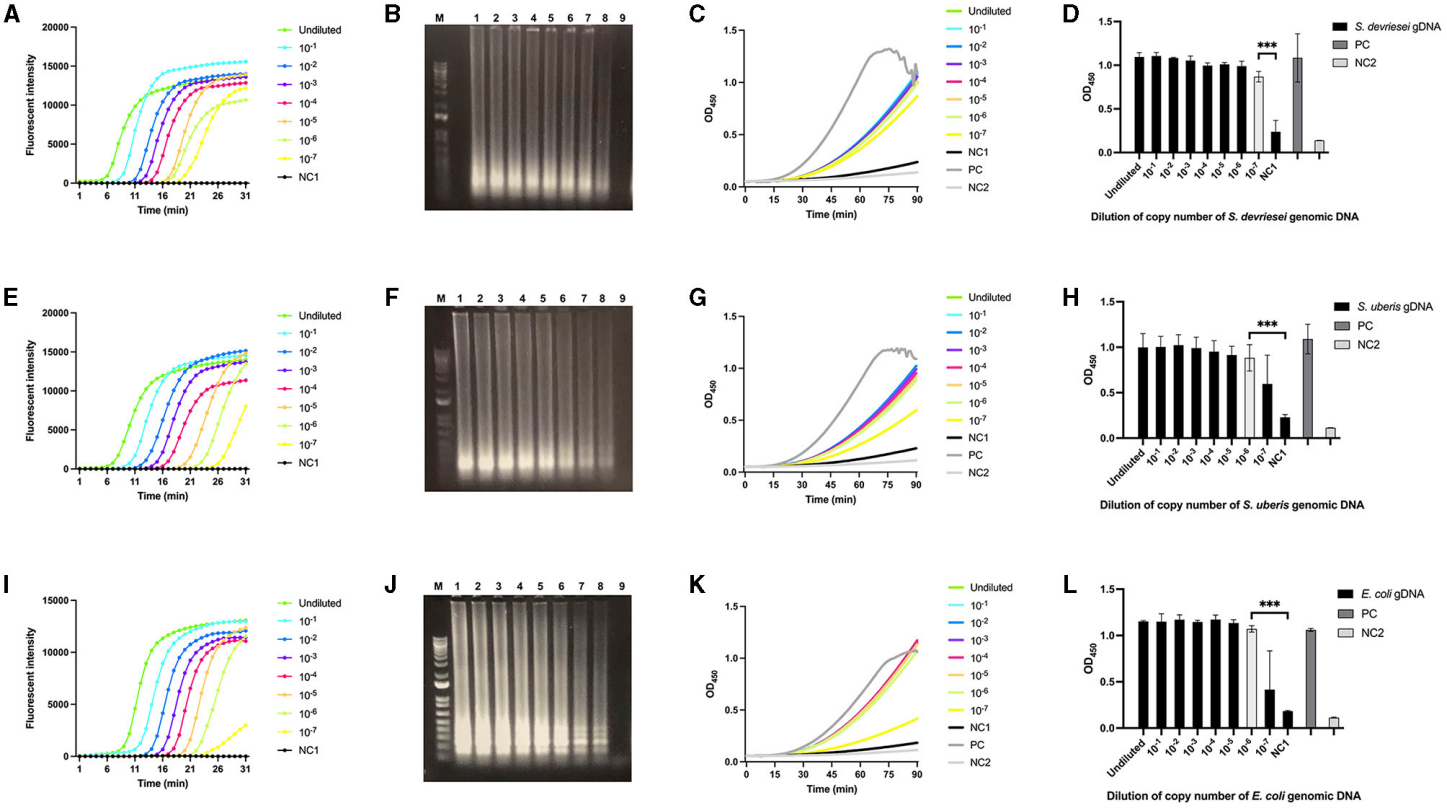
Limit of detection of genomic DNA from *S devriesei, S. uberis* or *E. coli* by LAMP followed by ABSTA. Fluorescent intensities in LAMP reaction were measured over time using various dilutions of bacterial gDNA copies: undiluted (bright green), and dilutions of 10 × (bright blue), 10^2^× (dark blue), 10^3^× (purple), 10^4^× (pink), 10^5^× (dark yellow), 10^6^× (light green), and 10^7^× (bright yellow) for *S. devriesei* by LAMPstaph **(A)**, *S. uberis* by LAMPstrep **(E)**, and *E. coli* by LAMPneg set **(I)**. DNase-free water served as the LAMP non-template control (NC1, black). Agarose gel electrophoresis showed ladder-like patterns of LAMP products from undiluted to 10^7^× diluted gDNA copies (lane 1 to 8) of *S. devriesei* **(B)**, *S. uberis* **(F)** and *E. coli* **(J)**, along with the LAMP non-template control (lane 9). The 1 kb Plus DNA ladder was utilized (lane M). OD_450_ values in ABSTA were measured in real-time utilizing LAMP products from *S. devriesei* **(C)**, *S. uberis* **(G)**, and *E. coli* **(K)** assays. The positive control (PC, dark gray) used double-stranded DNA dsFF_DG74, and the ABSTA non-template control (NC2, light gray) used DNase-free water. The OD_450_ values at 90 min in ABSTA showed the complementation of SpoIIID-C and N-SpoIIID proteins binding to LAMP products from *S. devriesei* **(D)**, *S. uberis* **(H)** and *E. coli* **(L)** assays (black bar), together with the positive control (dark gray bar) and ABSTA non-template control (light gray bar). Very significant between-group differences are shown by ***p < 0.001, where the limit of detection of ABSTA is indicated in white bars.

The gDNA copies in three LAMP assays using LAMPstaph, LAMPstrep, and LAMPneg primer sets were in the ranges 1.9 × 10^7^ to 1.9, 2.4 × 10^7^ to 2.4, and 1.0 × 10^7^ to 1.0, respectively. As expected, fluorescent intensity (FI) curves indicated a longer time before exponential amplification for lower copies within the 30-min reaction time. Non-template control gave no fluorescent signal, indicating that no primer secondary structure formed with three sets (Figures 3A, E, I). Agarose gel electrophoresis results were consistent with the LAMP assays, with band brightness decreasing with decreasing starting gDNA copies (Figures 3B, F, J). The 90-min ABSTA reactions utilizing the corresponding LAMP amplicons demonstrated real-time variations in OD_450_ values (Figures 3C, G, K). According to the endpoint OD_450_ values, the detection limits of ABSTA following LAMPstaph, LAMPstrep, and LAMPneg assays were determined to be 1.9, 24, and 10 gDNA copies in each LAMP assay, respectively (Figures 3D, H, L).

### 3.3 Detection limit of ABSTA following LAMP with DNA extracted from bacteria-spiked milk

**Figure 4:**
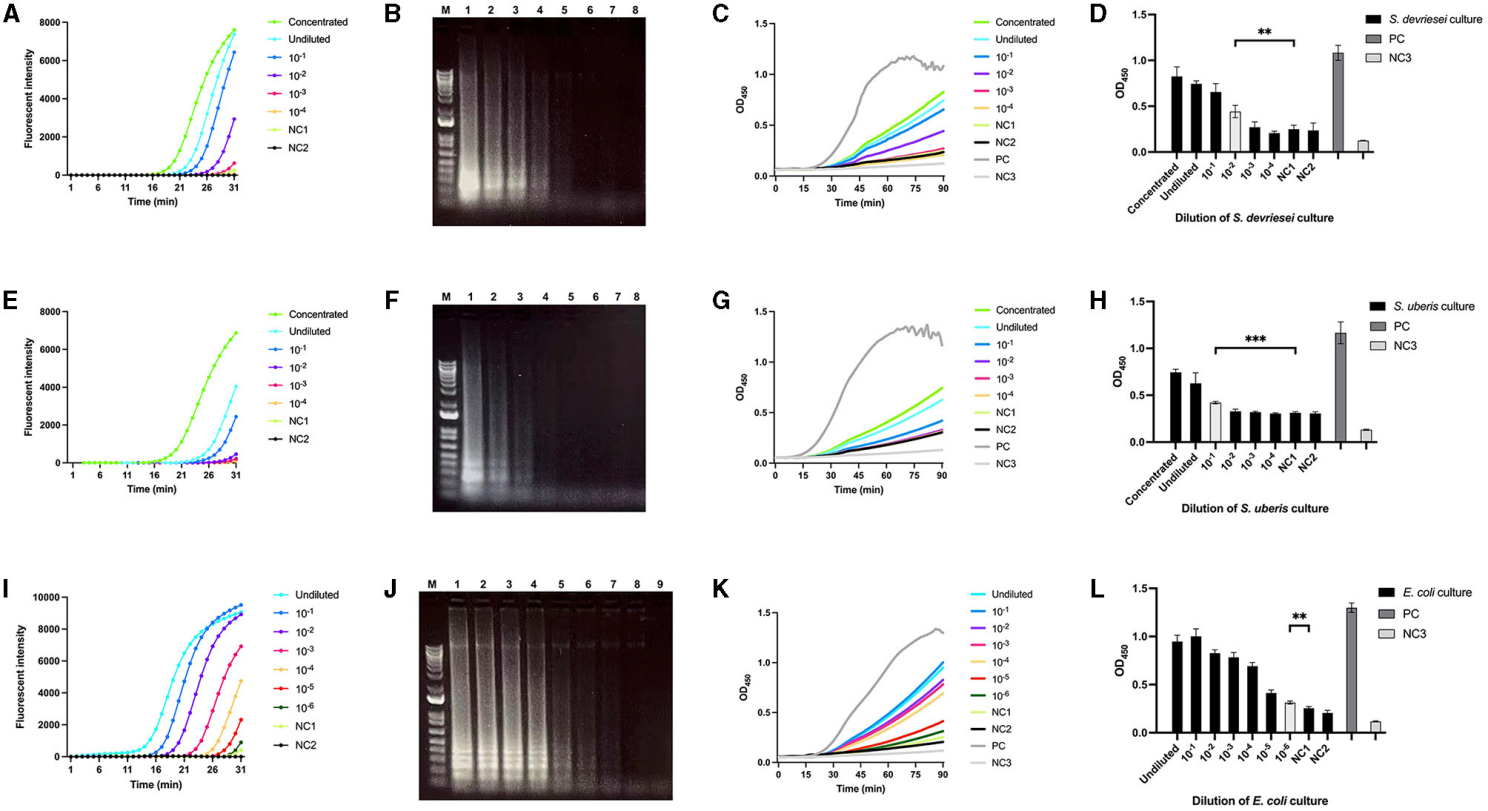
Limit of detection of bacterial DNA extracted from milk spiked with *S devriesei, S. uberis* or *E. coli* by LAMP followed by ABSTA. Fluorescent intensities in LAMP reaction were measured over time using DNA extracted from milk samples spiked with various concentrations of bacterial cells from cultures of OD_600_ = 1: 10 × concentrated (bright green), undiluted (bright blue) and dilutions of 10 × (dark blue), 10^2^× (purple), 10^3^× (pink) and 10^4^× (dark yellow) for *S. devriesei* by LAMPstaph **(A)**; *S. uberis* by LAMPstrep **(E)**; and additional 10^5^× (red) and 10^6^× (dark green) dilutions for *E. coli* by LAMPneg set **(I)**. Negative controls included DNA from PBS-spiked milk (NC1, light green) and DNase-free water (NC2, black) as the LAMP non-template control. Agarose gel electrophoresis showed ladder-like patterns of products, ranging from 10 × concentrated to NC2 assays (line 1 to 8) for LAMPstaph **(B)** and LAMPstrep **(F)** sets, and from undiluted to NC2 assays (line 1 to 9) for LAMPneg **(J)**. The 1 kb Plus DNA ladder was utilized (lane M). OD_450_ values in ABSTA were measured in real-time utilizing LAMP products from *S. devriesei* **(C)**, *S. uberis* **(G)**, and *E. coli* **(K)** assays. The positive control (PC, dark gray) used double-stranded DNA dsFF_DG74, and the ABSTA non-template control (NC3, light gray) used DNase-free water. The OD_450_ values at 90 min in ABSTA showed the complementation of SpoIIID-C and N-SpoIIID proteins binding to LAMP products from *S. devriesei* **(D)**, *S. uberis* **(H)** and *E. coli* **(L)** assays (black bar), together with the positive control (dark gray bar) and ABSTA non-template control (light gray bar). Very significant between-group differences are shown by ***p < 0.001 and **0.001 < *p* < 0.01, where the limits of detection of ABSTA are indicated in white bars.

The CFU/ml in milk samples spiked with *S. devriesei, S. uberis*, and *E. coli* ranged from 4.9 × 10^7^ to 4.9 × 10^2^, 2.0 × 10^7^ to 2.0 × 10^2^, and 1.1 × 10^7^ to 11, respectively. The fastest exponential curves were seen after 16th min for both LAMPstaph and LAMPstrep for the highest bacterial counts in milk, while LAMPneg only required 10 min to start exponentially amplifying DNA extracted at the same level of CFU/ml from *E. coli*-spiked milk (Figures 4A, E, I). Longer initiation time was needed as CFU/ml in each sample decreased. The flat curves of the PBS control and LAMP non-template control indicated that there was no secondary structure for the three primer sets. The variation in band brightness observed after electrophoresis between samples was consistent with the fluorescence signals (Figures 4B, F, J). The real-time ABSTAs employing the products of the three LAMP assays indicated that OD_450_ values began to increase after 25th to 30th min until the endpoint readouts were collected at 90th min (Figures 4C, G, K). The limits of detection of ABSTA corresponding to LAMPstaph, LAMPstrep, and LAMPneg primer sets were 4.9 × 10^4^ CFU/ml for *S. devriesei*, 2.0 × 10^5^ CFU/ml for *S. uberis*, and 11 CFU/ml for *E. coli*, respectively (Figures 4D, H, L).

Based on the *Ct* values of the multiplex real-time PCR with PathoProof™ Mastitis Kits, all specified CFU/ml levels in both *S. devriesei*- and *E. coli*-spiked milk samples were detected. For the most diluted level of *S. devriesei* at 4.9 × 10^2^ CFU/ml, the *Ct* value was 29, suggesting that the limit of detection for PathoProof™ Mastitis Kits in relation to *S. devriesei* is lower than 4.9 × 10^2^ CFU/ml. Similarly, the reaction for 11 CFU/ml of *E. coli* in spiked milk yielded a *Ct* value of 34, establishing the limit of detection for *E. coli* in spiked milk as 11. Furthermore, using the four build-in PCR primer mixes, the remaining PathoProof reactions showed true negative results with no false positive results for both bacteria. This indicated that the specificity of the kit was determined as 100% for this study.

### 3.4 Specificity of detection of SpoIIID recognition sequence-incorporated LAMP products

The gDNA of ten bacterial isolates was tested by LAMP assay LAMPstaph, LAMPstrep, and LAMPneg, at 10^6^ and 10^3^ copies per reaction (Table 2). At one million copies level, all the designated primer sets started exponential amplification of their specific target isolates only between the 8th to 13th min, except for the *Corynebacterium amycolatum*, which was amplified by LAMPneg at 23rd min. However, at the one thousand copies level, the initiation of exponential amplification was delayed till the 15th to 19th min by all the primer sets. The agarose gel electrophoresis results were consistent with the fluorescent signals (data not shown). The cross-reactivity of the LAMPstaph assay was determined to be 0%, with 4 true positive and 16 true negative results out of 20 tests. Similarly, the LAMPstrep assay also exhibited 0% cross-reactivity, with 6 true positive and 14 true negative results out of 20 tests. The LAMPneg assay demonstrated 8% cross-reactivity, with 8 true positive, 1 false positive, and 11 true negative results out of 20 tests. Therefore, the cross-reactivity of the three LAMP assays ranged from 0 to 8%.

**Table 2:** Cross-reactivity test on bacterial genomic DNA by three LAMP primer sets.

**Bacterial isolates**	**Primer sets**					
	**LAMPstaph**		**LAMPstrep**		**LAMPneg**	
	**Genomic DNA copy numbers per reaction**					
	**10** ^6^	**10** ^3^	**10** ^6^	**10** ^3^	**10** ^6^	**10** ^3^
*Staphylococcus devriesei* 41816325	10^*^	15	-	-	-	-
*Staphylococcus aureus* USA300	11	17	-	-	-	-
*Streptococcus uberis* 10501290	-	-	10	18	-	-
*Streptococcus dysgalactiae* 20304478	-	-	10	17	-	-
*Streptococcus uberis* 10107041	-	-	10	17	-	-
*Corynebacterium amycolatum* 40200255	-	-	-	-	23	-
*Escherichia coli* DH10β	-	-	-	-	8	17
*Escherichia coli* 10109298	-	-	-	-	10	17
*Klebsiella pneumoniae* 10116692	-	-	-	-	13	19
*Enterobacter hormaechei* 11104490	-	-	-	-	13	19

^*^Time to reach exponential amplification in minutes.

## 4 Discussion

Three Gram type-specific LAMP assays to detect DNA of the majority of bovine mastitis causing bacteria were developed to be compatible with the conditional complementation of split-trehalase recombinant proteins fused to the DNA binding protein SpoIIID. Three LAMP primer sets, LAMPstaph, LAMPstrep and LAMPneg, with specificity for *Staphylococcus spp., Streptococcus spp*. and Gram-negative bacteria, respectively, were designed based on the 16S rRNA genes of common bovine mastitis pathogens with the tandem SpoIIID specific recognition sequences introduced in the loop primers. The three sets of LAMP primers were subsequently used to amplify DNA extracted from bacterial cultures and spiked milk samples and detected by the ABSTA platform technology. With the previously optimized binding conditions of fusion proteins N-SpoIIID and SpoIIID-C (29), the limits of detection of ABSTA were established for both Gram-types. A satisfactory limit of detection was found for Gram-negative bacteria in milk samples, whereas it was found to be unsatisfactory for Gram-positives. High specificity of the three derived LAMP assays were demonstrated by amplifying gDNA from ten common isolates from both Gram-types of mastitis bacteria with expected signals originating from the restoration of trehalase activity by ABSTA. Combining LAMP and glucose signaling in this study holds promise for a fast and user-friendly POC diagnostic platform for informed bovine mastitis treatment decisions.

For LAMP primer design, we took advantage of the 16S rRNA gene consisting of highly conserved regions flanked by variable regions (34). Primers were designed for the conserved regions of the 16S rRNA genes with sufficient differences between Gram-positive and Gram-negative bovine mastitis bacteria, while minimizing the number of primers required for the entire assay. This approach was chosen over LAMP assays targeting either genus or species-specific genes, such as the *nuc* or *PhoA* genes specific for detecting *S. aureus* and *E. coli* in bovine mastitis, respectively, due to the reduced labor in experimental preparation and shorter time-to-result for the pathogenic Gram-type identification (35, 36).

The combined method relies on split trehalase complementation enabled by the specific recognition of a consensus sequence by the DNA-binding protein SpoIIID to detect LAMP amplicons. SpoIIID was selected as the DNA-binding protein of choice because its binding to DNA as a monomer is critical with regards to avoiding self-complementation of the fusion proteins (30). According to multiple studies, split TreA is capable of protein complementation by recombination of the TreA fragments fused to analyte-sensing elements (25–28). Based on these characteristics, the combination of split TreA platform and SpoIIID can be used to detect the amplicons incorporated with tandem SpoIIID specific recognition sites, indicating presence or absence through the glucose signals generated by the conditional recovery of the trehalase capacity. The extended loop primers of the initialized three primer sets were critical to obtain endpoint concatemer products containing large amounts of the target of our fusion protein reagents, because the loop primers simultaneously introduced the specific sequences and boosted the LAMP reaction. Interestingly, the presented research is one of very few studies relying on modifying LAMP loop primers for enhanced amplicon detection (37–39).

In comparison with the tests on genomic DNA, the limit of detection of the LAMP reaction for bacteria-spiked milk by the LAMPstaph and LAMPstrep based assays was greatly impacted. This might be partly due to difficulties in lysing Gram-positive bacteria cell wall to release sufficient target DNA. Compared with Gram-negatives, the peptidoglycan layer on the cell wall of Gram-positive bacteria is thicker and more extensively cross-linked (40, 41). Despite the integration of the prescribed bead-beating procedure for the mechanical breakdown of the bacterial cell wall, the LAMP reactions of Gram-positive bacteria were still delayed when applied to DNA extracted from spiked milk (42). However, this presumed challenge of DNA extraction from Gram-positives milk samples was not supported by the results from the PathoProof™ multiplex real-time PCR. Likely other unidentified factors were at play with the LAMP assays for Gram-positives. The PathoProof test exhibited a limit of detection more than a hundred times lower than the combined LAMP-ABSTA on milk samples spiked with *S. devriesei*, while maintaining the same level for *E. coli*. Nonetheless, the high-priced single reaction and necessity for a thermal cycling system and analysis software restrict this commercialized real-time PCR from widespread adoption and the potential to be developed as a POC method.

The LAMP assays with three modified primer sets successfully demonstrated high specificity of discriminating common bovine mastitis pathogens. *Corynebacterium amycolatum* was unexpectedly amplified by LAMPneg at 10^6^ copies, but the maximum bacterial count for mastitis milk caused by this organism is < 10^5^ CFU/ml. So, it is unlikely that the associated gDNA levels in infected milk would be detected by LAMPneg primer set in practical applications (43). Therefore, the specificity of the three proposed LAMP primer sets for detecting *Staphylococcus, Streptococcus*, and Gram-negative bacteria gDNA remains reliable. Besides the specificity provided by the 16S rRNA-based primer design, additional specificity came from the ABSTA-recognition sequences in the loop primers. Similar to a qPCR probe, the requirement for the loop primer to recognize an internal sequence to participate in the reaction enhanced the specificity. Based on mastitis pathogen prevalence studies, this combined LAMP-ABSTA method would be able to correctly identify the Gram-type of 86%−96% of the intramammary infections on dairy farms in a much shorter time than when using conventional bacterial culturing or PCR (6, 44, 45).

Antibiotics are widely used in the dairy industry for mastitis control (46). Nonetheless, there exists a pressing need for more informed and precision-oriented therapeutic approaches in addressing the challenges of antimicrobial resistance (AMR) and tailored drug administration of Gram-negative cases (47, 48). The introduced LAMP-ABSTA platform offers promising detection limits and specificity for both Gram-types based on genomic DNA, along with demonstrated high sensitivity for Gram-negative infected milk. Significant potential persists for this platform to decrease the detection limit for Gram-positive cocci in infected milk, particularly through further enhancements in DNA extraction methods. With reduced time in distinguishing Gram-types, this platform enables a faster antimicrobial treatment which potentially reduces the need for broad-spectrum antibiotics. This also reduces the change of transmission within the healthy herd in case of contagious pathogens, further limiting the risk of AMR spreading to herd mates. The methodology unquestionably contributes to the effective implementation of Gram-type specific antimicrobial strategies aimed at reducing multidrug resistance while concurrently maintaining or elevating standards of animal welfare (2, 49). Furthermore, existing LAMP assays for other target sequences of interest, such as antimicrobial resistance genes, can be modified by mere integration of SpoIIID recognition sequences into loop primers. This expands the applicability of this platform to the precise diagnosis of causative bacteria not only in the bulk milk with less pre-processing but also in various contagious diseases and their AMR genes.

The potential for contamination of samples during the milk sampling process is an important aspect to consider with respect to the performance of this methodology. Avoiding contamination is undoubtedly crucial when testing for mastitis pathogens using both culture and molecular methods of detection. In the case of culture, the presence of more than two types of colony morphologies is indicative of a contaminated sample. However, this criterion does not apply to molecular methods. Our presented novel method could indeed be affected by contaminating bacteria. This impact will require further investigation by testing a substantial number of clinical samples, including those verified as contaminated by culture. To mitigate this problem, a somatic cell count (SCC) measurement could be conducted in parallel to confirm the presence of mastitis and the likelihood of an intramammary infection to prevent unnecessary treatment. Selective treatment decision of clinical mastitis cases can be made based on an evidence-based protocol taking into account a combination of rapid diagnostic test results, review of somatic cell count and clinical mastitis records (8). Inhibitory substances can also affect the performance of LAMP assays on DNA extracts from clinical milk samples. Compared to healthy milk, the mastitic milk matrix presents a more complex composition, with abnormally altered levels of components including somatic cells, proteins, fat, and ions, which can potentially compromise both DNA extraction and downstream molecular amplification. Therefore, it is essential to further investigate the sensitivity and specificity of the proposed LAMP assay method using milk samples from clinical cases.

While the proposed methodology performed well in detecting target pathogens, its current implementation necessitates a relatively time-consuming ABSTA, advanced instruments and skilled personnel, particularly for large-scale sample testing. To enable on-farm use, simplification of the presented methodology could be achieved by customizing the LAMP assay reagents and implementation. For instance, LAMP reagents can retain activity at room temperature by lyophilization or sucrose stabilization (50, 51). More ideally, integrating the proposed ABSTA and LAMP into a closed tube technique would not only enhance the convenience of on-farm testing but also mitigate false positive results due to cross-contamination of LAMP products (52). Remarkably, the LAMP operating temperature can be further reduced from 65°C to 40°C by applying phosphorothioate modification to LAMP inner primers, while maintaining similar sensitivity (53). These findings offer promising opportunities to enhance this proposed methodology as a straightforward, time-efficient, and highly cost-effective field-compatible diagnosis method.

Positive LAMP signal is typically visualized with the SYBR dye, turbidimeter or UV light, presenting certain limitations for on-site testing (54, 55). Lateral flow technology was also demonstrated to have diagnostic potential to detect LAMP amplicons (23). In contrast, our ABSTA testing approach results in glucose readouts, which are stable and highly compatible with commercially available handheld glucometers to help shorten the time-to-results, as demonstrated in a previous study (29). Therefore, the introduced split-enzyme detection technology provides a reliable and accessible means for LAMP signal transduction, providing an improved solution for on-site quantification of target bacteria. While this work has not yet yielded a ready-to-use POC method, ongoing research and development efforts, including refinements to the ABSTA method, are expected to result in further advancements within this area.

## Data availability statement

The original contributions presented in the study are included in the article/supplementary material, further inquiries can be directed to the corresponding author.

## Author contributions

ZM: Data curation, Formal analysis, Investigation, Methodology, Writing – original draft. JDB: Conceptualization, Funding acquisition, Project administration, Resources, Supervision, Validation, Writing – review & editing.
